# Abnormal Large-Scale Network Activation Present in Bipolar Mania and Bipolar Depression Under Resting State

**DOI:** 10.3389/fpsyt.2021.634299

**Published:** 2021-03-26

**Authors:** Can Zeng, Brendan Ross, Zhimin Xue, Xiaojun Huang, Guowei Wu, Zhening Liu, Haojuan Tao, Weidan Pu

**Affiliations:** ^1^Department of Psychiatry, The Second Xiangya Hospital, Central South University, Changsha, China; ^2^The China National Clinical Research Center for Mental Health Disorders, Changsha, China; ^3^McGill Faculty of Medicine, Montreal, QC, Canada; ^4^Medical Psychological Institute, The Second Xiangya Hospital, Central South University, Changsha, China

**Keywords:** bipolar mania, bipolar depression, group independent component analysis, resting state fMRI, brain image

## Abstract

**Introduction:** Previous studies have primarily focused on the neuropathological mechanisms of the emotional circuit present in bipolar mania and bipolar depression. Recent studies applying resting-state functional magnetic resonance imaging (fMRI) have raise the possibility of examining brain-wide networks abnormality between the two oppositional emotion states, thus this study aimed to characterize the different functional architecture represented in mania and depression by employing group-independent component analysis (gICA).

**Materials and Methods:** Forty-one bipolar depressive patients, 20 bipolar manic patients, and 40 healthy controls (HCs) were recruited and received resting-state fMRI scans. Group-independent component analysis was applied to the brain network functional connectivity analysis. Then, we calculated the correlation between the value of between-group differences and clinical variables.

**Results:** Group-independent component analysis identified 15 components in all subjects, and ANOVA showed that functional connectivity (FC) differed significantly in the default mode network, central executive network, and frontoparietal network across the three groups. Further *post-hoc t*-tests showed a gradient descent of activity—depression > HC > mania—in all three networks, with the differences between depression and HCs, as well as between depression and mania, surviving after family wise error (FWE) correction. Moreover, central executive network and frontoparietal network activities were positively correlated with Hamilton depression rating scale (HAMD) scores and negatively correlated with Young manic rating scale (YMRS) scores.

**Conclusions:** Three brain networks heighten activity in depression, but not mania; and the discrepancy regions mainly located in prefrontal, which may imply that the differences in cognition and emotion between the two states is associated with top–down regulation in task-independent networks.

## Introduction

Bipolar disorder (BD) is a common severe psychiatric disorder with two opposite states, depression, and mania, which can occur in repeating or alternating episodes. Patients experiencing a manic episode are optimistic and show an increase in goal-directed activity (1), whereas those in a depressive episode are downhearted, pessimistic, and oriented toward internal thoughts (2–5), implying that different neuropathological underpinnings likely produce these overtly clinical distinctions. Thus, the identification of pathophysiologic biomarkers that differ between bipolar depression and bipolar manic can both inform dynamic change of BD and provide biological targets for the development of personalized treatments.

Previous studies have often performed emotional face-recognition tasks to examine the neuropathological mechanisms driving mania and depression (6–13), which have particularly involved a so-called “emotional circuit” that comprised the limbic structures (such as the amygdala, insula, anterior cingulate cortex) and prefrontal cortex including the medial prefrontal cortex (mPFC) and orbital frontal cortex (OFC) (14–18). Frontal hypofunction, for example, may provide a shared neural basis for mania and depression, and studies have consistently found decreased frontal activation or hypoconnectivity across mood states during tasks (11, 19–21), suggesting weakened top–down regulation. However, the amygdala, a key facet of emotional processing, exhibits inconsistent activation or functional connectivity (FC) that varies across the experimental paradigms and BD phases (7, 8, 11, 14, 22), with some studies finding increased amygdala activity, whereas others have found decreased activity. Research has suggested that the highs and lows of amygdala activity may be a state-dependent phenomenon caused by trait-like hypoactivation of regulatory frontal regions in BDs (9), but others have proposed that the amygdala fluctuations were affected by medication use or different emotional recognition tasks (e.g., sad, happy, fear face) (11).

These task-based functional magnetic resonance imaging (fMRI) studies have lent deep insight into the neuropathology of BD, particularly the role of an abnormal corticolimbic circuit; however, constrained by the limited emotion activation that one task can probe, our understanding of BD-related brain functional alteration remains fragmented. Fortunately, resting-state fMRI offers a unique opportunity to inspect the brain-wide functional architecture. To date, studies have characterized the neuropathology of manic or depressive states separately, but very few studies have used resting-state fMRI to examine differences or changes between the two states (1, 23–25). What should be noted is that these resting-state fMRI studies have mostly focused on circumscribed regions, such as the amygdala or striatal to mapping the abnormal brain function connectivity. Only one study examined the abnormal topographical balance between two specified networks (1). However, the brain cannot be fully understood by topographical characterization alone as it works as a complex system of interacting subsystems; therefore, mapping the whole brain large-scale networks may help to identify any difference in abnormality between mania and depression and thus will deepen our understanding of BD.

In this study, by applying a holistic brain-wide functional connectivity analysis of resting-state fMRI data, we aimed to investigate the different neural correlates of bipolar mania and depression in the resting state. This study compared the neuroimaging findings between bipolar mania, bipolar depression, and healthy controls (HCs) using the method of group-independent component analysis (gICA). Group-independent component analysis can separate independent “sources,” mainly those in the spatial dimension that have been mixed, and as a result, it resists the skewing of results due to researchers' assumptions (26). Additionally, the relationship between the blood oxygen level-dependent (BOLD) signal of intrinsic networks (INs) and clinical scales, namely, the Hamilton Depressive Rating Scale (HAMD) and Young Mania Rating Scale (YMRS), was explored as well.

## Materials and Methods

### Subjects

The patients including 41 bipolar depressive patients and 20 bipolar manic patients were recruited from outpatient and inpatient settings; most of the manic (18/20) and depressive subjects (31/41) were recruited from inpatient settings, whereas the remainder were outpatients. All patients were currently experiencing an episode and met the diagnostic criteria for the Structured Clinical Interview–Patient Version of the *Diagnostic and Statistical Manual of Mental Disorders, Fourth Edition* (SCID-I/P). Forty-two age- and sex-matched HCs were recruited from the community and met the diagnostic criteria of SCID Non-Patient confirming that neither them nor their first-degree relatives had any psychiatric disorders. The inclusion criteria for all the subjects were as follows: (a) aged 18–60 years, (b) right-handed, (c) received at least 9 years of education [due to the influence of education level on the brain function (27)]. And for the bipolar depressive patients, the HAMD score was ≥17, and YMRS score ≤6; and for bipolar manic patients, the YMRS score was ≥12, and HAMD score ≤7. Exclusion criteria included (a) other psychiatric disorder or significant physical illness, personality disorder, intellectual disability or substance dependence (except tobacco); (b) recent electric shock treatment; and (c) alcohol or benzodiazepines taken 24 h before the interview and fMRI.

All the patients were recruited at the Second Xiangya Hospital of Central South University, were provided information about the procedures, and signed the informed consent. The study was approved by the ethics committee of Xiangya.

### Scan Acquisition and Data Preprocessing

All fMRI data were obtained using a Philips Gyroscan Achieva 3.0-T scanner (36 slices, repetition time = 2,000 ms, matrix = 64 × 64, echo time = 30 ms, flip angle = 90°, slice thickness = 4 mm, gap = 0 mm). Each subject was scanned 250 volumes, and the whole brain was effectively covered. When scanning, subjects were asked to close their eyes and remain calm without excessive thinking. Head motion was strictly restricted; motion beyond 2.5 mm was discarded, and seven subjects' data were excluded for this reason. We also performed “scrubbing” to make sure that head-motion artifacts did not influence the observed effects. An assessment of head motion at each time point was computed as the frame-wise displacement (FD). Consistent with previous studies (28), any image with FD >0.5 was discarded and replaced with a linear interpolation. The mean absolute FD across the three groups did not differ significantly [mean HC: 0.145, (SD = 0.053), mean bipolar depression: 0.144 (SD = 0.079), mean bipolar mania: 0.181 (SD = 0.102)]. The data were preprocessed by DPARSF (29). The first 10 volumes were removed to allow scanner calibration and subjects' adaptation to the environment, and the remaining 240 volumes were processed by SPM8 (University College London, UK; http://www.fil.ion.ucl.ac.uk/spm). During preprocessing, the images were spatially normalized to a standard template (Montreal Neurological Institute) and resampled to 4 × 4 × 4-mm voxels, with standard parameters adopted throughout the preprocessing. Then, images were smoothed with an isotropic Gaussian kernel, the full-width at half maximum = 8 mm. Finally, the processed data were further temporally bandpass filtered (0.01–0.08 Hz) and linearly detrended to reduce the influence of low-frequency drifts and physiological high-frequency noise.

### Independent Component Analysis

Spatial ICA was directed with resting-state fMRI data from all 103 participants using the Informix algorithm with the Group ICA of the fMRI Toolbox (GIFT) software (Medical Image Analysis Lab, University of New Mexico, Albuquerque, NM, USA; http://icatb.sourceforge.net/) (29); this exact model has also been used in our prior work (30–32). According to the regular minimum description length criteria tool, 35 spatial independent components (ICs) were determined. With principal component analysis, dimensions of the functional data were then reduced (26, 33), followed by an IC assessment by the Informix algorithm that generated spatial maps and time courses. Assessed ICs at the group level were then back-reconstructed for each participant established in principal component analysis compression and projection (26, 34), and each estimated component received subject-specific spatial maps and time courses. This specific back-reconstruction of the GIFT algorithm permits simultaneous analysis of all of the participants as part of a large ICA group matrix (34). Therefore, for each IC, the time courses of each component represented a pattern of synchronized brain activity, whose coherency pattern across voxels was represented in the related spatial map. And to exhibit voxels relevant to a particular IC, the intensity values were converted to *z* values in each map.

### Identifying Resting State Networks

To identify valid resting state networks, the standard method of rejecting artifacts was adopted. First, an experienced researcher examined the network components visually to eliminate those clearly representing artifacts and then correlated spatially with a priori probabilistic gray-matter, white-matter, and cerebrospinal-fluid (CSF) templates using multiple regression in SPM8. Components having high association (|β| > 2) with CSF and white matter and low association (|β| < 0.5) with gray matter were removed (35). Finally, 20 components were removed as noise, and 15 valid components remained (see the Supplementary Materials Figure 1S). To make sure that only highly correlated brain regions were analyzed, we employed an explicit mask created by a voxel-wise one-sample *t*-test (*p* < 0.05 with family wise error (FWE) correction at voxel level).

### Statistical Analysis

The SPM8 software was applied to the statistical analysis on fMRI data. After valid ICs were identified, by including the age as the covariate, one-way analysis of covariance (ACOVA) was performed with group as the control variable and BOLD signal of each IC as the observable variable to compare the FC differences among three groups, which is masked with the results from one-sample *t*-tests. An initial statistical threshold was set at *p* < 0.05 with FWE correction at the cluster level (*k* > 40) and uncorrected *p* < 0.001 at the voxel level. Further pairwise comparisons, including age as the covariate as well, were conducted using the mask involving the regions with significant differences in ACOVA. The statistical threshold for significance was also set at *p* < 0.05 with FWE correction at the cluster level (*k* > 40) and uncorrected *p* < 0.001 at the voxel level. By using REST (http://resting-fmri.sourceforge.net) (36), we extracted the BOLD signals of ICs exhibiting significant differences across the three groups with YMRS scores and HAMD scores using Pearson correlation (*p* < 0.05), including age as covariate. Considering bipolar depression and bipolar mania are two internally related and continuum-like states of one disorder, we merged two patient groups together to compute correlation.

## Results

### Demographic and Clinical Characteristics

Demographic and clinical characteristics of the three groups are shown in Table 1. There is no significant difference in age, sex, age at illness onset, or illness duration across the three groups. Given that age produced marginal significance and antipsychotic use produced significant difference between patient groups, we included them as covariates in further analysis.

**Table 1:** Subjects demographic and clinical characteristics.

	**BD-D (*n* = 41)**	**BD-M (*n* = 20)**	**HC (*n* = 42)**	***F*/χ^**2**^/*t***	***P***
Age, mean (SD) (years)	26.7(7.11)	28.1(8.13)	24.07(4.8)	2.793	0.066
Male	17	7	22	1.614	0.446
Female	24	13	20		
YMRS	1.95	23.7	—	−19.61^**^	0.00
HAMD	20.8	4.2	—	13.76^**^	0.00
Unmedicated (*n*)	7/41	4/20		0.078	0.519
Antipsychotics (*n*)	22/41	15/20		36.83^**^	0.00
Duration at onset (months)	55	73.8	—	2.335	0.316
Age at onset (years)	22.1	22.05	—	0.726	0.95

*BD-D, bipolar depression; BD-M, bipolar mania; HC, healthy control; YMRS, Young Manic Rating Scale; HAMD, Hamilton Depression Scale*.

* *P < 0.05*,

** *P < 0.01*.

### Distinct Regions From Intrinsic Networks

**Figure 1:**
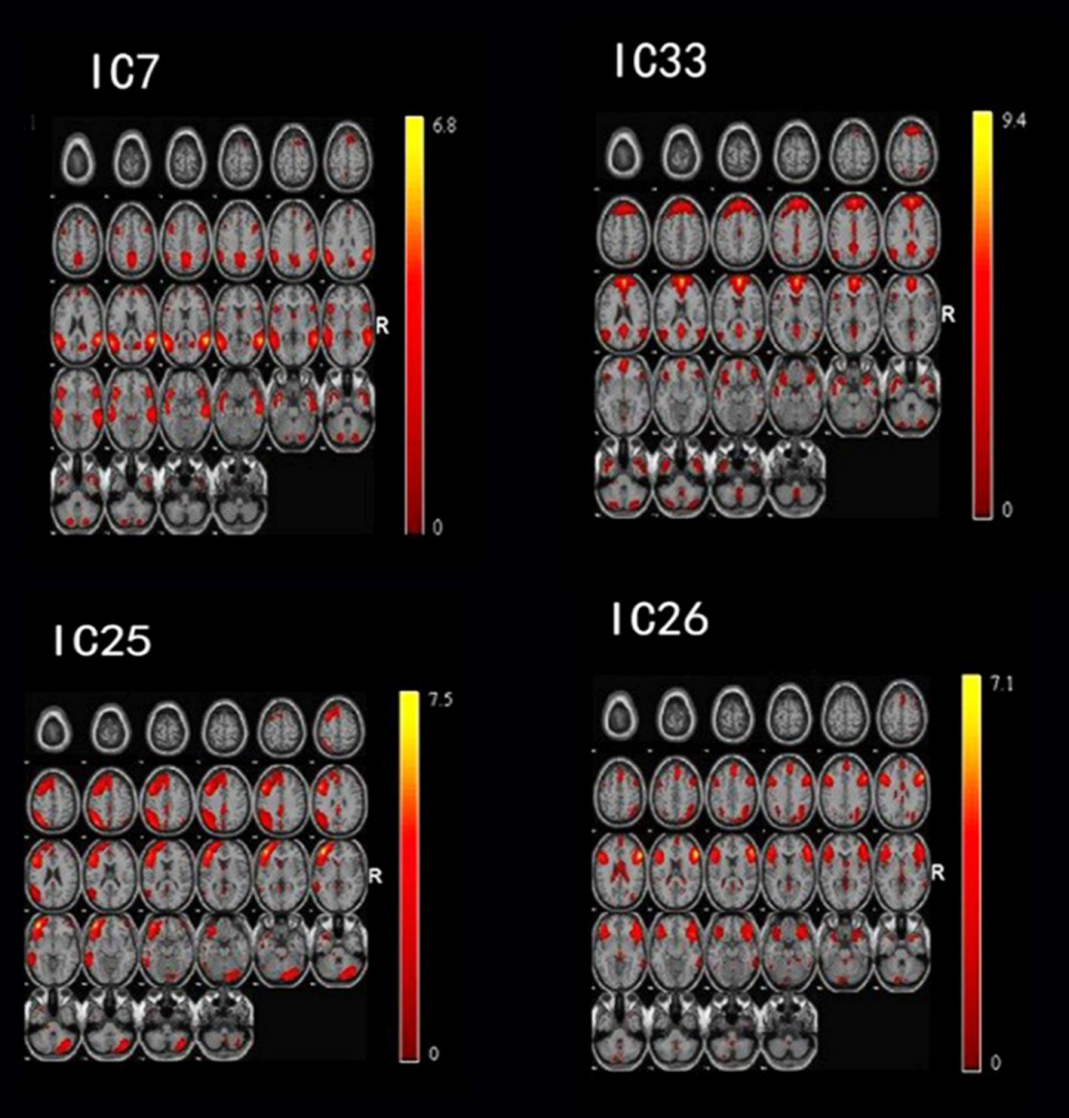
By using the method of gICA, three intrinsic networks, including the default mode network (DMN) located at the IC7 and IC33, frontoparietal network (FPN) located at the IC25, and central executive network (CEN) located at the IC26, showed significant activity differences across healthy controls, bipolar depressive and bipolar manic patients.

**Figure 2:**
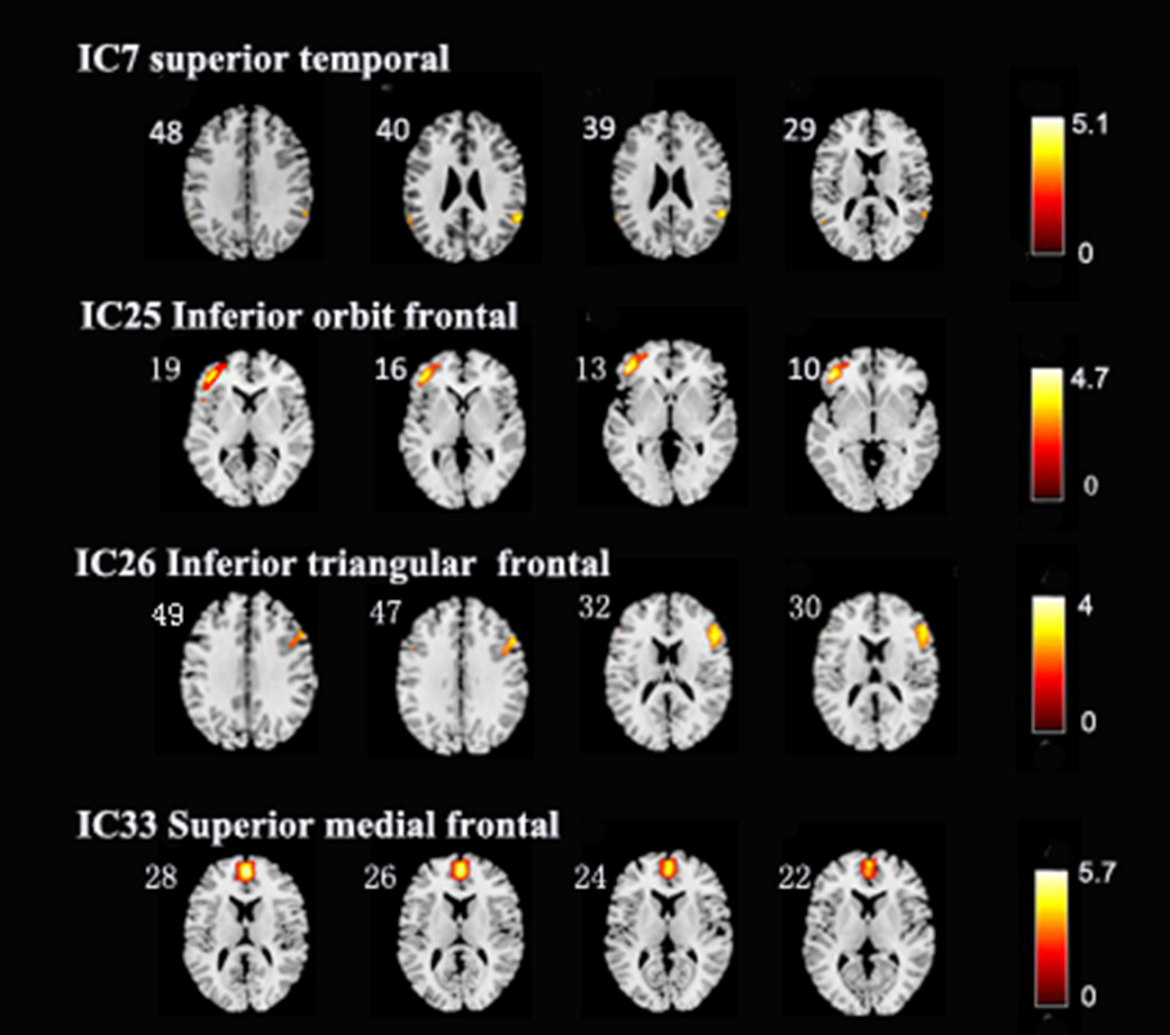
In the three intrinsic networks, four regions, namely, inferior orbit frontal gyrus (L) in frontoparietal network (IC25), triangle inferior frontal gyrus(R) in central executive network (IC26), superior temporal (R), and frontal superior medial gyrus (L) in the default mode network (IC7, IC33) showed significant activity discrepancy across three groups (FWE, *p* < 0.05 corrected).

After performing one-way ANOVA analysis across the three groups for the remaining 15 valid ICs, three INs, including the DMN (IC7, IC33), frontoparietal network (FPN) (IC25), and central executive network (CEN) (IC26), showed significant differences (Figure 1). The locations with significant differences in each network were the left orbit inferior frontal gyrus for IC25 (Figure 2), right triangle inferior frontal gyrus for IC26, and the left frontal superior medial gyrus and right superior temporal for the DMN (IC7 and IC33), respectively (Table 2).

**Table 2:** Distinct regions extracted from brain intrinsic networks.

**IC**	**AAL region (L/R)**	**Voxel size**	**MNI coordinate (*x,y,z*)**
IC7 (default mode network)	Superior temporal (R)	41	54, −51, 21
IC33 (default mode network)	Frontal superior medial gyrus (L)	80	−6, 45, 45
IC25 (frontoparietal network)	Orbit inferior frontal (L)	77	−45, 36, −15
IC26 (central executive network)	Triangle inferior frontal (R)	126	54, 24, 15

*R, right, L, left; IC, independent component; MNI, Montreal Neurological Institute; AAL, anatomical automatic labeling*.

Further *post-hoc* testing found that the activity of these four regions showed a decline in activity trending across the three groups (depression > HC > mania). Except for the difference between the mania group and HC (at an uncorrected *p* < 0.001 level), differences of FC within four regions between depression and HC, as well as between depression and mania, all survived after FWE correction (*p* < 0.05) (Table 3).

**Table 3:** *post-hoc* t-testing of the four brain regions.

**Brain region**	**BD-D VS. BD-M**	**BD-D VS. HC**
(IC25) orbit inferior frontal gyrus (L)	*t* = 5.15, *P*_FWE_ = 0.011^*^	*t* = 4.61, *P*_FWE_ = 0.048^*^
(IC26) triangle inferior frontal gyrus (R)	*t* = 5.34, *P*_FWE_ = 0.003^**^	*t* = 4.91, *P*_FWE_ = 0.009^**^
(IC33) frontal superior medial gyrus (L)	*t* = 5.35, *P*_FWE_ = 0.005^**^	*t* = 4.55, *P*_FWE_ = 0.062
(IC7) superior temporal (R)	*t* = 4.72, *P*_FWE_ = 0.028^*^	*t* = 4.57, *P*_FWE_ = 0.035^*^

*BD-D, bipolar depression, BD-M, bipolar mania, HC, healthy control; L, left; R, right*.

* *P < 0.05*,

** *P < 0.01*.

### Correlation Between Intrinsic Networks and Clinical Features

Correlation analysis showed that the BOLD signal of the FPN has significant positive association with HAMD score (*r* = 0.486, *p* < 0.01) and significant negative correlation with YMRS (*r* = −0.552, *p* < 0.01). Similar correlation was found for CEN, positive association with HAMD score (*r* = 0.535, *p* < 0.01), and significant negative correlation with YMRS (*r* = −0.578, *p* < 0.01), but no significant correlation was found with the DMN.

## Discussion

This study aimed to explore the distinct alterations of large-scale brain networks between bipolar manic and depressive phases and found that there were three large-scale networks, including the DMN, FPN, and CEN, presenting a gradient of declining activity from depression to mania, with higher activity in depression and lower activity in mania (depression > HC > mania). Further, the BOLD signal of the FPN and CEN both presented a positive correlation with HAMD, but a negative correlation with YMRS.

The DMN, including the mPFC, precuneus, posterior cingulate cortex, inferior parietal cortex, and hippocampus, has attracted extensive attention in this field (37, 38). This network is suggested to activate in the resting state and is involved in self-referential and introspective thought (39–41). Researchers had consistently found that it maintained hyperconnectivity in depressive patients (32, 42), but was inconsistently activated in mania, showing decreased connectivity or no significant difference (43, 44). Interestingly, this study found that the DMN, particularly the mPFC, displayed increased activity in bipolar depressive patients, but decreased in bipolar manic patients. The mPFC has been proposed to subserve attentional modulation on self-relevant information such as recalling autobiographical memory and episodic future thought related to oneself (45–47); therefore, a possible mechanism for the DMN being implicated in BD may be best explained by understanding from depression and mania, respectively. Patients in the depressive episode may be consumed by overly pessimistic thoughts about themselves in the past and future, which relates to hyperfunction of the DMN. In contrast, patients in the manic episode are easily drawn from their own attention to external environmental irrelevant stimuli instead of self-relevant stimuli, finally leading to the decreased activation of the DMN.

The FPN showed significantly greater activation discrepancy among the three groups. Earlier studies suggested that the FPN is associated with cognitive and executive control processes during goal-directed behavior (48–50), However, other studies also showed that the FP engages in self-referential tasks (i.e., recollecting one's past or imagining one's personal future), coupled with the DMN functioning as a mediator (51). Further, it has been documented that BD patients in depressive episodes often perform mental work to defensively ward off negative cognition about themselves (52), such as avoiding feelings of failure and uselessness; thus, overactivation of the FPN possibly serves a compensatory role in cognition for depressive patients. Anatomically, the OFC is a core region of the prefrontal cortex and is one of the regions most tightly connected with the amygdala and other subcortical limbic structures relevant to emotion (53, 54), implicating its role in emotion regulation. Numerous studies have shown that the metabolism, neurons, glial cell density, and gray matter volume of the OFC have significant alterations in major depressive disorder (MDD) (55–57). In particular, effective treatment for MDD often decreases the hyperfunction of OFC (58), suggesting that greater activation of the OFC may lead to excessive inhibition of emotion. In contrast, mania patients show decreased activation of the OFC or ventrolateral prefrontal (VLPFC) during the resting state (14, 59), which has been proposed to result in decreased inhibition of emotion in mania patients. Therefore, dysfunction of the OFC may lead to inappropriate emotional responses to changes in internal stimuli and environmental contexts (59), ultimately resulting in manic or depressive episodes.

The CEN also shows activation discrepancy across the three groups in the right triangular inferior frontal gyrus. The network is mainly composed of multiple prefrontal cortex regions and the lateral posterior cingulate cortex, which shows strong cooperative activation in a wide range of cognitively demanding tasks (60), actively participating in maintaining and managing information in working memory, solving rule-based problems, and making decisions in goal-directed behavior (61). Furthermore, some studies found that the CEN plays an important role in processing task-related information and suppressing the interference of irrelevant information (62). Deficits in these processes are characteristic of many major mental disorders and neurological disorders including BD, MDD, schizophrenia, and autism—they have all displayed dysfunction of the CEN (63, 64). In this network, we found that the triangular inferior frontal gyrus was abnormally activated in both states; thus, this region is suggested to be pertinent to reactive inhibition. Clinical studies from attention-deficit/hyperactivity disorder or substance-dependence patients with reactive inhibition deficits showed right inferior frontal cortex dysconnectivity during inhibitory tasks (64). Furthermore, a study also found that the degree of depression significantly correlated with the activation of the inferior frontal gyrus during a Go/No-Go task (65), suggesting that the inferior frontal gyrus affects the individual's level of cognitive inhibition in depression. Similar to the findings of OFC in the FPN relevant to the emotional suppression, this study also found increased IFG activation in depressive patients but decreased IFG activation in mania patients, suggesting the excessive cognition inhibition (such as memory deficit and lower thought speed) in depression and deficient cognition inhibition in mania (such as distractibility and racing thoughts). Consistent with this notion, our further partial correlation analysis found that greater activity in both FPN and CEN related to more severe depressive symptoms, whereas lower activity in two networks related to more severe manic symptoms.

Several limitations should be noted in the present study. First, medication may have been a confounding factor in our findings. Nearly all patients in the study were taking medications; mood stabilizers were used by most patients, and other medications (such as antidepressants, antipsychotics, and benzodiazepines) were also used according to patients' clinical performance. Future studies in drug-naive BD patients are warranted to verify the findings in our study. Second, the differences of the three networks between the bipolar mania patients and HCs did not survive after multiple corrections in our study, which may be due to the relatively small sample size of mania patients; thus, our findings on the decreased activity in three large-scale brain networks in mania patients should be replicated in future studies with larger sample size. Third, the study is a cross-sectional design, which limited us to explore the dynamic changes between depression and mania in BD patients, and further longitudinal studies with medical free patients are needed to clarify the dynamic transformation.

## Conclusions

We compared whole-brain networks across bipolar depression and bipolar manic patients and HCs and by using the method of gICA, we found differences mainly in the core intrinsic brain networks across the DMN, FPN, and CEN. The discrepancy between bipolar mania and bipolar depression may be associated with top–down regulation in task-independent networks, which may underlie the differences in cognition and emotion between the two states.

## Data Availability Statement

The original contributions presented in the study are included in the article/Supplementary Material, further inquiries can be directed to the corresponding author/s.

## Ethics Statement

The studies involving human participants were reviewed and approved by The ethics committee of Xiangya: beisha tang, huihuan tang, xiaoxia zuo, long mo, yuexiang lv, yixiong li, haihe jiang, jianping ning, shifang peng, meizuo zhong, yi shen, lu shen, yinglan li, wenen liu, runhua li, and tao yao. The patients/participants provided their written informed consent to participate in this study.

## Author Contributions

CZ wrote the main manuscript text, BR and ZL edited language, WP and ZX designed the experiments. XH and GW helped to analyzing data. HT revised the manuscript. All authors contributed to the article and approved the submitted version.

## Conflict of Interest

The authors declare that the research was conducted in the absence of any commercial or financial relationships that could be construed as a potential conflict of interest.
