# Supplementary figures and images for: Abnormal Large-Scale Network Activation Present in Bipolar Mania and Bipolar Depression Under Resting State
Source: Front Psychiatry. 2021 Mar 26;12:634299. doi: 10.3389/fpsyt.2021.634299 (PMC8032940; doi:10.3389/fpsyt.2021.634299)

**
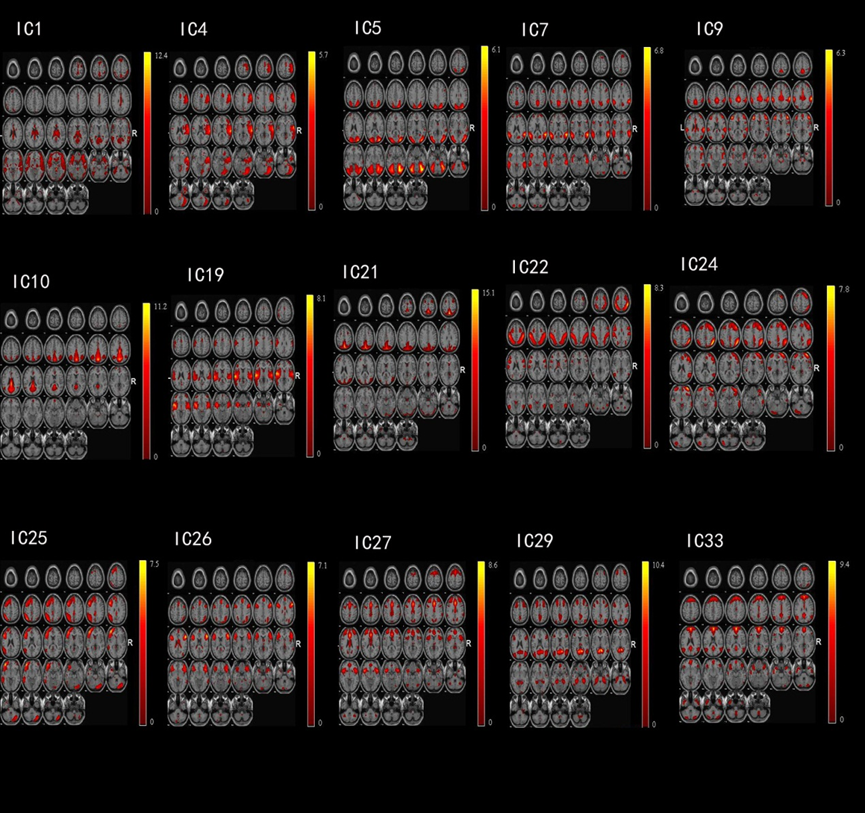
**

**Fig.1s 15 valid components left after discarding artifacts.**

Supplement: Supplementary file 1 [file Table_1.DOCX]
